# Effects of Tafamidis Meglumine on Transient Focal Neurological Episodes and Meningeal Contrast Enhancement in Hereditary Transthyretin‐Related Meningeal Amyloidosis: Report of Two Patients Carrying the c.265T>C (p.Y89H) Variant

**DOI:** 10.1002/brb3.70856

**Published:** 2025-09-10

**Authors:** Natsumi Saito, Yasuko Kuroha, Ayaka Ishiyama, Takahiro Wakasugi, Takayoshi Tokutake, Arika Hasegawa, Tetsuya Takahashi

**Affiliations:** ^1^ Department of Neurology NHO Nishiniigata Chuo Hospital Niigata Japan

**Keywords:** hereditary ATTR amyloidosis, tafamidis meglumine, TFNEs, Y69H

## Abstract

**Background:**

Y69H (p.Y89H) variant hereditary transthyretin (ATTRv) amyloidosis causes meningeal amyloidosis, with mutant TTR deposits localized to the leptomeninges and vitreous body.

**Methods:**

The effect of tafamidis meglumine on neurological disorders, such as the frequency of transient focal neurological episodes (TFNEs), magnetic resonance imaging (MRI) findings, and TTR levels in cerebrospinal fluid, was investigated in two patients diagnosed with Y69H ATTRv mutation.

**Results:**

The initial symptoms in both patients were TFNEs, such as aphasia, sensory disturbance, motor paralysis, ataxia, and drop attacks. Neither epileptic drugs nor antiplatelet therapy decreased the frequency of attacks. The patients exhibited diffuse leptomeningeal enhancement on brain and spinal MRI. Tafamidis meglumine was initiated at a dose of 20 mg/day and was found to be partially effective. The TFNEs nearly resolved, and meningeal enhancement on brain MRI improved; however, the neurological deficits progressed over the following 2 years.

**Conclusions:**

Tafamidis had a partial effect on TFNEs and meningeal contrast enhancement on MRI; however, cerebellar ataxia and cognitive decline continued to progress.

## Graphical abstract

We evaluated the efficacy of tafamidis to two patients with Y69H (p.Y89H) variant hereditary transthyretin amyloidosis presenting with significant TTR deposits in the meninges. The results showed that the frequency of transient focal neurological episodes decreased and that MRI meningeal contrast enhancement improved, while cerebellar ataxia, cognitive decline, and superficial siderosis progressed. ASM; anti‐seizure medicine, ope; vitreous surgery.

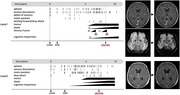

## 1 Introduction

Leptomeningeal amyloidosis is a rare phenotype of hereditary transthyretin (ATTRv) amyloidosis that presents with transient focal neurological episodes (TFNEs) and progressive cognitive dysfunction. The c.265T>C, p.Y89H (Y69H) mutation is one of 17 reported cases to date (Qin et al. 2021; Laura et al. 2021). Few reports have described the effect of disease‐modifying therapy for ATTRv meningeal amyloidosis because the meningeal subtype is rare and the diverse presentation of TFNEs often makes diagnosis challenging. Two cases of patients affected by the Y69H variant belonging to different families originating from the northern region of Niigata prefecture in Japan are reported here.

## 2 Case 1

**FIGURE 1:**
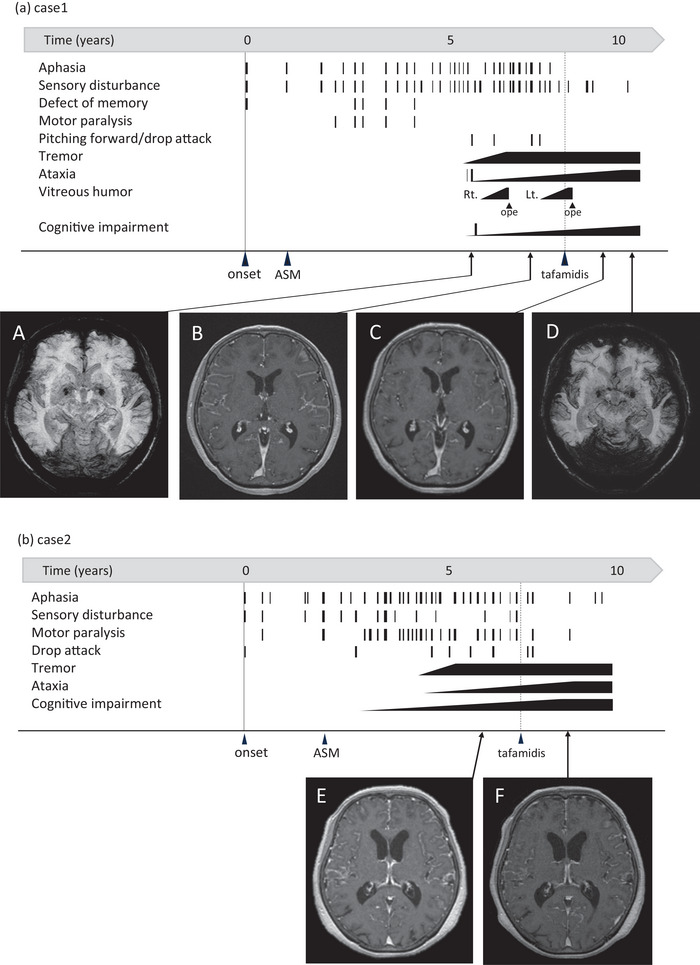
Clinical course, treatment, and changes in MRI findings of the two cases. Frequency of TFNEs and progressive symptoms in Cases 1 (a) and 2 (b). More than 5 years after symptom onset, both cases showed contrast enhancement of the meninges on Gd‐T1WI MRI. Case 1 also showed superficial iron deposition on SWI, which was among the key findings supporting the diagnosis of meningeal amyloidosis. In both cases, tafamidis treatment was started approximately 7 years after disease onset, resulting in a marked reduction in TFNEs. Meningeal enhancement on Gd‐T1WI decreased after the start of treatment in both cases (images B–C and E–F), while in Case 1, progression of low‐signal area on SWI was observed, suggesting hemosiderin deposition on the brain surface (images A–D). ASM, antiseizure medication; ope, vitreous surgery.

**FIGURE 2:**
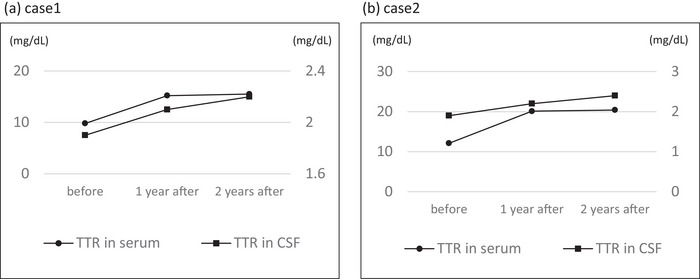
Changes in transthyretin concentration in serum and cerebrospinal fluid before and after tafamidis treatment. Transthyretin concentration in both serum and cerebrospinal fluid increased in both cases for up to 2 years after the initiation of tafamidis treatment.

A 62‐year‐old Japanese woman developed transient numbness in her right leg and difficulty in speaking at the age of 53 years (Figure 1a). She had a family history of cerebellar ataxia, tremors, epilepsy, and superficial siderosis. Her plain brain magnetic resonance imaging (MRI) and magnetic resonance angiography (MRA) were normal. The patient was diagnosed with a transient ischemic attack. Since her subsequent transient aphasia lasted 2–3 days, the patient was referred to our epilepsy center. She was commenced on 500 mg/day of levetiracetam orally, which was increased to 1000 mg in the same year. Initially, the frequency of neurological episodes was once every 2–3 months. However, in the fourth year after onset, this increased to two to three times per month. The dose of levetiracetam was increased to 2000 mg, but was ineffective. Long‐term video electroencephalography monitoring during transient neurological episodes revealed no paroxysmal discharges. At the age of 58, she developed a subarachnoid hemorrhage and was referred to our neurological department. The patient exhibited both motor and sensory aphasia during the episode, which was characterized by difficulty finding words (anomia), literal paraphasia, and impaired comprehension of spoken sentences. These symptoms were not accompanied by impaired consciousness or syncope. After recovering from the transient attack, slight cerebellar truncal ataxia persisted. A brain MRI was performed using a 1.5‐T scanner. Susceptibility‐weighted images (SWI; image A in Figure 1) were acquired with a repetition time (TR) of 8 ms and echo time (TE) of 46 ms. Axial T1‐weighted gadolinium‐enhanced images (Gd‐T1WI; image B in Figure 1) were obtained with TR/TE of 60/4.2. SWI revealed linear low‐signal areas over the entire brain surface (image A), and diffuse leptomeningeal enhancement was prominent on Gd‐T1WI (image B). A comprehensive diagnostic evaluation was conducted in this patient presenting with enhanced leptomeninges and cerebral hemosiderosis. Initial laboratory tests, including levels of serum antineutrophil cytoplasmic antibody (ANCA), antinuclear antibody, and angiotensin‐converting enzyme (ACE), were within normal ranges. No bleeding tendencies were observed. MRA showed no evidence of dural arteriovenous malformation, thrombosis, or aneurysm. Cerebrospinal fluid (CSF) analysis revealed normal cell counts, ACE, and soluble interleukin‐2 receptor levels. Given the clinical presentations, amyloid angiopathy was considered a potential diagnosis. Further genetic analysis revealed a c.265T>C (p.Y89H) mutation in the transthyretin (*TTR*) gene. The patient's episodes were identified as TFNEs attributable to amyloid angiopathy. After the administration of tafamidis meglumine (20 mg/day), she had fewer TFNEs over the following 2 years. The meningeal enhancement on Gd‐T1WI MRI decreased after the start of treatment (images B and C), whereas progression of the low‐signal area on SWI was observed, suggesting hemosiderin deposition on the brain surface (images A–D). Both serum and CSF levels of transthyretin were elevated (from 9.8 to 15.2 and 15.5 mg/dL in serum, and from 1.9 to 2.1 and 2.2 mg/dL in CSF, measured before treatment, and at 1 and 2 years after administration, respectively) (Figure 2a). Although TFNEs decreased following tafamidis administration, cerebellar ataxia progressively worsened over time, correlating with the increasing hemosiderin deposition observed on SWI. Cognitive decline also gradually worsened (as detailed in Table S1), with intermittent fluctuations. Notably, these neurological deteriorations and the progression of superficial siderosis occurred despite the reduction in meningeal enhancement on Gd‐T1WI after tafamidis treatment.

## 3 Case 2

A 74‐year‐old Japanese woman with transient logopenic aphasia and motor paralysis was diagnosed with leptomeningeal amyloidosis associated with Y69H ATTRv mutation, based on cerebrospinal MRI, genetic analysis, and histopathological examination (Figure 1b; Saito et al. 2023). She had a family history of tremors and falls and was diagnosed with Lewy body dementia. Her first symptom, at the age of 66, was a transient falling episode with aphasia. The frequency of neurological episodes, lasting from 30 min to 2 h, was once every 2–3 months. Over the following 3 years, the frequency of episodes increased to once or twice per month. She was administered 2000 mg of levetiracetam orally with 800 mg of valproic acid. Five years after onset, the episodes lasted as long as 3 days. Perampanel, lacosamide, and lamotrigine were tested in sequence but were ineffective. She developed tremors and limb and truncal ataxia, which gradually progressed. Gd‐T1WI and SWI MRI scans were acquired under the same conditions as those in Case 1. Unlike in Case 1, SWI did not reveal superficial hemosiderosis. Brain and whole spinal cord MRI showed diffuse meningeal contrast effects that were particularly strong at the level of the lower thoracic spinal cord. A thorough assessment was performed to investigate the leptomeningeal enhancement observed in this patient. Diagnostic tests were performed for malignant meningitis, fungal infection, tuberculosis, toxoplasmosis, ANCA‐associated vasculitis, sarcoidosis, rheumatoid meningitis, and other collagen vascular diseases; however, all results were negative. After starting tafamidis meglumine (20 mg/day), the patient's TFNE frequency decreased to two episodes in 2 months and to only three episodes in the subsequent 2 years, similar to Case 1. Despite a reduction in meningeal enhancement on Gd‐T1WI following tafamidis administration (images E and F), cognitive decline, including logopenic aphasia, progressed significantly and irreversibly (Table S2). As dementia advanced, cerebellar ataxia became increasingly difficult to evaluate, whereas intention tremors, which were prominent prior to tafamidis treatment, remained unchanged. In contrast to Case 1, no temporal progression of hemosiderin deposition was observed on SWI. Both serum and CSF levels of transthyretin increased from 12.1 to 20.1 and 20.4 mg/dL in serum and from 1.9 to 2.2 and 2.4 mg/dL in CSF, before treatment and 1 and 2 years after administration, respectively (Figure 2b).

## 4 Discussion

Common features of these two cases were contrast enhancement of the leptomeninges and leakage of gadolinium into the subarachnoid space on Gd‐T1WI, which decreased after the administration of tafamidis. Enhancement of the meninges generally reflects blood‐brain barrier (BBB) dysfunction. In particular, in the leptomeningeal form, the deposition of mutated TTR compromises the BBB from a pathological perspective (Nakamura et al. 2005; Chao et al. 2025). A reported autopsy case with enhanced leptomeninges revealed extensive amyloid deposition in the subarachnoid small arteries, intraparenchymal small arteries, and parenchyma of the spinal cord and brain (Nakamura et al. 2005). Causal TTR is produced in the choroid plexus, where tafamidis has very low permeability under normal conditions (Monteiro et al. 2018). It is likely that tafamidis could pass through the barrier and stabilize the tetramers because it is impaired by the deposition of mutated TTR. In fact, tafamidis decreased the frequency of TFNEs and reduced leptomeningeal enhancement on MRI in the two cases (Figure 1). Furthermore, the concentration of transthyretin in the CSF increased following tafamidis administration in both cases (Figure 2). Based on findings in these two cases, increased levels of transthyretin may be reflected in reduced deposition in the leptomeninges. Indeed, it has been demonstrated that levels of stabilized transthyretin tetramers increase in the CSF of patients with ATTRv following tafamidis administration (Monteiro et al. 2018; Tsai et al. 2023). Therefore, transthyretin concentration in the CSF may serve as a useful biomarker for evaluating treatment efficacy. However, it should be noted that the dissociation rate of TTR subunits could not be measured, and the stabilizing effect of tafamidis at the molecular level could not be directly assessed. Furthermore, previous studies (Monteiro et al. 2018) compared small cohorts of treated and untreated patients but did not examine longitudinal changes before and after tafamidis administration. These limitations should be considered when interpreting the findings of this study.

In Case 1, the absence of epileptic discharges during TFNEs, as confirmed by video electroencephalogram (EEG) monitoring, may have aided in the differential diagnosis of epilepsy. TFNEs typically do not exhibit epileptic discharges and show limited responsiveness to antiseizure medications. The mechanism underlying TFNEs is hypothesized to involve localized seizures, cortical spreading depression, or focal vasospasm triggered by the accumulation of blood breakdown products in the subarachnoid space and superficial cortical layers (Charindimou et al. 2012). The resulting electrical activity may be too subtle to detect as an abnormal scalp EEG finding.

Cerebellar ataxia and cognitive impairment gradually progressed despite improvement in the contrast effect of the pia mater and decreased frequency of TFNEs (Figure 1). We speculate that these symptoms may arise through a mechanism different from that of TFNEs. As accumulated transthyretin amyloid cannot be removed by tafamidis, it is thought that functional impairment progresses as it gradually accumulates (Ziskin et al. 2015). To overcome these problems, high‐dose administration or other disease‐modifying therapies may represent viable treatment options. Superficial iron deposition is also a refractory problem. Iron chelation therapy may be an effective treatment approach for patients with confirmed hemosiderin deposition on SWI MRI. In the case of meningeal amyloidosis, further development of therapeutic agents with sufficient efficacy to treat central nervous system lesions is required. To support patients with chronic diseases such as ATTRv, especially considering the variable clinical responses observed in the two presented cases, it is important to implement regular monitoring and promote multidisciplinary collaboration. Longitudinal periodic assessments play a key role in tracking disease progression and evaluating treatment efficacy over time.

## 5 Conclusion

Tafamidis partially decreased the frequency of TFNEs and contrast enhancement in two patients with the TTR Y69H variant of hereditary ATTR amyloidosis; however, cerebellar ataxia and cognitive impairment gradually progressed.

## Author Contributions

**Natsumi Saito**: writing–original draft. **Yasuko Kuroha**: writing–review and editing. **Ayaka Ishiyama**: supervision. **Takahiro Wakasugi**: supervision. **Takayoshi Tokutake**: supervision. **Arika Hasegawa**: supervision. **Tetsuya Takahashi**: writing–review and editing, supervision, project administration.

## Conflicts of Interest

The authors declare no conflicts of interest.

## Peer Review

The peer review history for this article is available at https://publons.com/publon/10.1002/brb3.70856.

## Supporting information




**Supplementary Table**: brb370856‐sup‐0001‐Table.pptx

## Data Availability

The data that support the findings of this study are openly available in [repository name e.g., “figshare”] at doi.org/[doi], reference number [reference number].
