# Supplementary material for: Effects of Tafamidis Meglumine on Transient Focal Neurological Episodes and Meningeal Contrast Enhancement in Hereditary Transthyretin‐Related Meningeal Amyloidosis: Report of Two Patients Carrying the c.265T>C (p.Y89H) Variant
Source: Brain Behav. 2025 Sep 10;15(9):e70856. doi: 10.1002/brb3.70856 (PMC12423105; doi:10.1002/brb3.70856)
Supplement: Supplementary file 1 — Supplementary Table: brb370856‐sup‐0001‐Table.pptx [file BRB3-15-e70856-s001.pptx]

## Slide 1
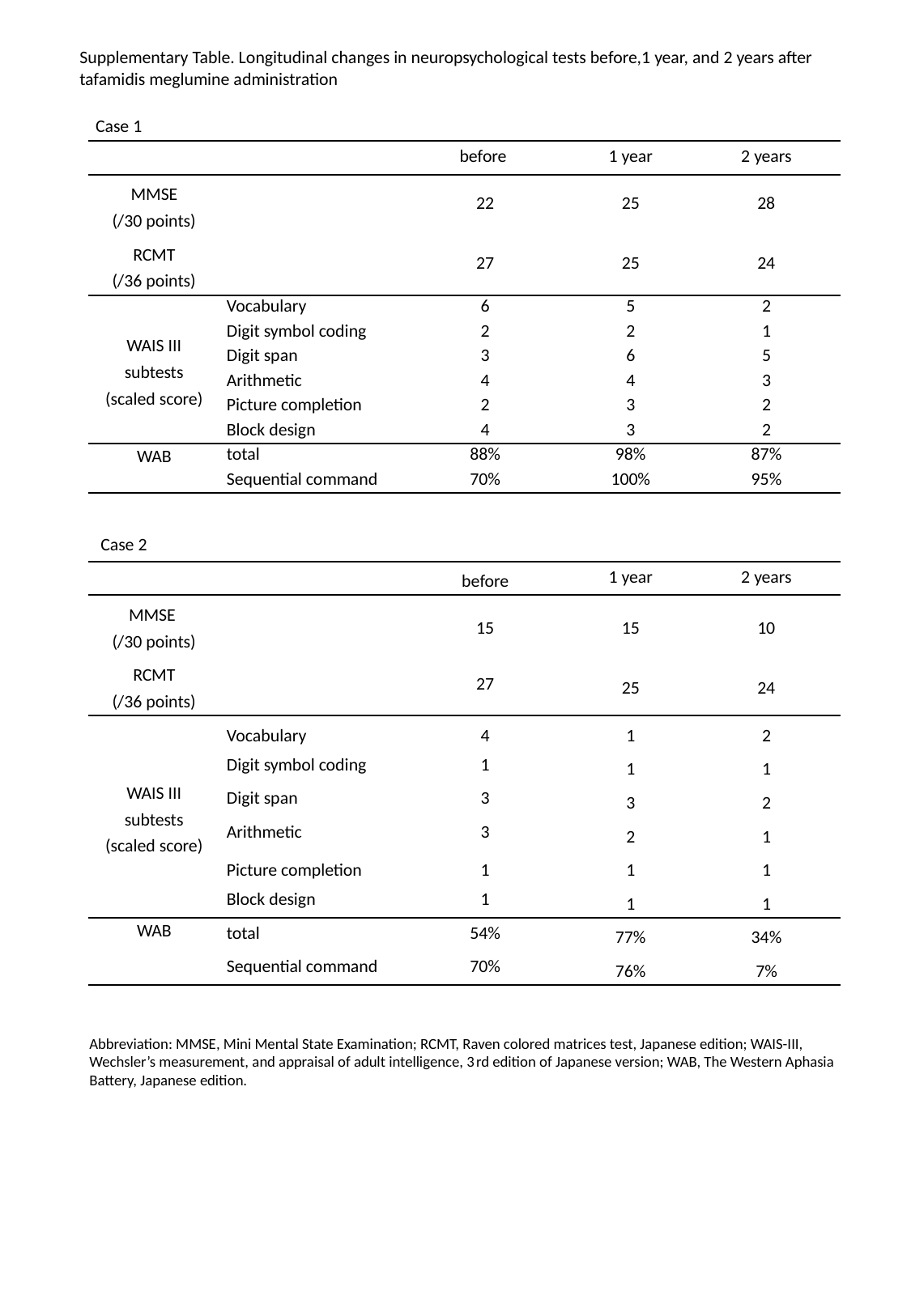

Supplementary Table. Longitudinal changes in neuropsychological tests before,1 year, and 2 years after tafamidis meglumine administration
| Case 1 | | | | |
| --- | --- | --- | --- | --- |
| | | before | 1 year | 2 years |
| MMSE (/30 points) | | 22 | 25 | 28 |
| RCMT (/36 points) | | 27 | 25 | 24 |
| WAIS III subtests (scaled score) | Vocabulary | 6 | 5 | 2 |
| | Digit symbol coding | 2 | 2 | 1 |
| | Digit span | 3 | 6 | 5 |
| | Arithmetic | 4 | 4 | 3 |
| | Picture completion | 2 | 3 | 2 |
| | Block design | 4 | 3 | 2 |
| WAB | total | 88% | 98% | 87% |
| | Sequential command | 70% | 100% | 95% |
| | | | | |
| Case 2 | | | | |
| | | before | 1 year | 2 years |
| MMSE (/30 points) | | 15 | 15 | 10 |
| RCMT (/36 points) | | 27 | 25 | 24 |
| WAIS III subtests (scaled score) | Vocabulary | 4 | 1 | 2 |
| | Digit symbol coding | 1 | 1 | 1 |
| | Digit span | 3 | 3 | 2 |
| | Arithmetic | 3 | 2 | 1 |
| | Picture completion | 1 | 1 | 1 |
| | Block design | 1 | 1 | 1 |
| WAB | total | 54% | 77% | 34% |
| | Sequential command | 70% | 76% | 7% |
Abbreviation: MMSE, Mini Mental State Examination; RCMT, Raven colored matrices test, Japanese edition; WAIS-III, Wechsler’s measurement, and appraisal of adult intelligence, 3 rd edition of Japanese version; WAB, The Western Aphasia Battery, Japanese edition.
